# Comparison of aflibercept and bevacizumab in the treatment of type 1 retinopathy of prematurity

**DOI:** 10.1186/s40942-021-00334-4

**Published:** 2021-10-13

**Authors:** Hamid Riazi-esfahani, Alireza Mahmoudi, Mehdi Sanatkar, Afsar Dastjani Farahani, Fatemeh Bazvand

**Affiliations:** Eye Research Center, Farabi Eye Hospital, Tehran University of Medical Science, Tehran, Iran

**Keywords:** Retinopathy of prematurity, Bevacizumab, Aflibercept

## Abstract

**Background:**

To evaluate the outcome of intravitreal bevacizumab (IVB) and aflibercept (IVA) injection for patients with retinopathy of prematurity (ROP).

**Methods:**

In this single-center retrospective cohort, the recorded medical data of the infants who had been undergone intravitreal injection with either bevacizumab or aflibercept for type 1 ROP were reviewed. The infants were allocated into two groups. IVB group included patients who were treated with bevacizumab as initial treatment and the IVA group included patients who were treated with aflibercept as initial treatment. The rate and time of complete regression, as well as the recurrence rates, were compared between the groups.

**Results:**

A total of 889 eyes of 453 infants were enrolled in the study. There were 865 eyes of 441 infants in the IVB group and 24 eyes of 12 infants in the IVA group. Follow-up time was 289 ± 257 days in the IVB group and 143 ± 25 days in the IVA group (p < 0.001). The difference in the ROP zone was not statistically significant between the 2 treatment groups (p = 0.328). All eyes in the IVA group showed initial regression of ROP after the intravitreal injections. These regressions were achieved in 830 (96.0%) eyes that were injected with IVB (p = 0.023). The median observed regression time was 10 days and 16 days in eyes treated with bevacizumab and aflibercept respectively. Recurrence was noted in 3.9% of eyes (34/865) in the IVB group and 58.3% of eyes (14/24) in the IVA group (p < 0.001).

**Conclusion:**

While the regression rate in the IVA group was significantly higher than in the IVB group, the recurrence rate was significantly more in the IVA group, which may be attributed to differences in the pharmacokinetics of these drugs in the vitreous body.

## Background

Retinopathy of prematurity (ROP) still is one of the leading causes of permanent visual loss worldwide [1]. Developing in premature children healthcare, and lack of proper ROP screening programs have resulted in high disease prevalence in the developing countries [2].

Dysregulation of vascular endothelial growth factor (VEGF) that leads to abnormal vasculogenesis as well as neovascularization is one of the causative factors for ROP development [3]. Indeed, ROP is a biphasic disease consisting of an initial phase of oxygen-induced vascular abolition followed by a period of hypoxia-induced vessel proliferation due to VEGF rise [4].

For many years, ablative therapies especially laser therapy was the preferred treatment for infants with ROP. These treatments destroy the peripheral avascular retina to regress the neovascularisation by decreasing the production of VEGF [5]

Some investigations have shown encouraging treatment outcomes after intravitreal bevacizumab (IVB) as a first-line treatment especially for Zone I ROP and aggressive posterior ROP (APROP) [6, 7]. In contrast to ablative therapies, anti-VEGF therapy is associated with a lower rate of high myopia and peripheral visual field loss, although it needs a longer intense follow-up schedule [2, 8].

It is worth knowing that anti-VEGF therapy is included in most of the ROP guidelines worldwide, Among them, bevacizumab is a complete antibody drug, which only binds to VEGF-A [6, 9]. Aflibercept (Eylea®, Regeneron) is a soluble decoy receptor, produced by fusing VEGF receptors 1 and 2 to the Fc portion of human immunoglobulin G1, which allows it to bind to all isoforms of VEGF-A, VEGF-B, and placental growth factor (PGF) [10].

In comparison with bevacizumab, aflibercept has a longer duration and more potent action due to higher binding affinity for VEGF [10].

Few studies compared the effects of intravitreal aflibercept (IVA) with other anti VEGFs in the treatment of type 1 ROP affecting either zone 1 or 2 [9, 11]. The objective of the present study is to compare the efficacy of bevacizumab and aflibercept treatments for pre-threshold type 1 ROP. We aimed to evaluate these intravitreal agents in terms of the treatment effect, disease regression, and recurrence profiles.

## Methods

This was a single-center retrospective cohort on the comparison of IVB (Avastin; Genentech, Inc., South San Francisco, CA) and IVA (Eylea®, Regeneron Pharmaceuticals Inc., Tarrytown, NY, USA) in the treatment of pre-threshold type 1 ROP. The study was conducted between 2012 and 2019 in the Farabi eye hospital, ROP center, working as a tertiary center for the screening and treatment of ROP in Iran. The institutional review board/ethics committee of the Tehran University of Medical Science approved this survey (https://ethics.research.ac.ir/IR.TUMS.FARABI.REC.1399.040). The infants’ parents or legal guardians provided informed consent. The study adhered to the principles laid out in the Declaration of Helsinki.

The recorded medical data of the infants who had been undergone intravitreal injection with either bevacizumab or aflibercept for type 1 ROP were reviewed. Type 1 ROP was defined by the criteria outlined in the Early Treatment for Retinopathy of Prematurity study: zone I with any stage with plus disease, zone I with stage 3 without plus disease, or zone II with stage 2 or 3 with plus disease.

Patients who had been received any previous treatments (laser, cryotherapy, and surgery) before intravitreal injections were excluded. Also, patients with anterior or posterior segment disorders or congenital anomalies (such as congenital cataracts or glaucoma) were excluded.

Intravitreal injections:

We use bevacizumab as a routine for all neonates with ROP who are candidates for intravitreal anti-VEGF therapy. In a period, due to the unavailability of bevacizumab, we preferred to use aflibercept instead of bevacizumab for intravitreal injections. The off-label use of bevacizumab for intravitreal injection in ophthalmology and aflibercept in ROP treatment was explained to parents. The intravitreal injections were performed in the operating room after parents signed an informed consent document.

Intravitreal injections were performed under topical anesthesia. Both eyes, when necessary, were treated at the same time with different vials. The periocular region was disinfected with 10% povidone-iodine, 5% povidone-iodine was instilled on the ocular surface, then half of the adult doses of bevacizumab (0.625 mg/0.025 mL) or aflibercept (1 mg/0.025 mL) was injected with a 30-gauge needle 1–1.5 mm behind the limbus into the vitreous cavity. Topical gentamycin or sulfastamide was given for 3 days postoperatively.

Patients were followed with indirect ophthalmoscopy by an expert retina specialist on the first day after injection and then weekly until the regression of ROP was documented which described as a prominent decrease in plus disease, clarity of the vitreous and, disappearance of the neovascularisations, after that every 2–3 weeks until full vascularization, which was described as reaching of the vascularization to the temporal Ora serrata without any active component, including tractional tissues, retinal detachment, or hemorrhage. On each visit, the infants were assessed in terms of regression or progression of plus disease and vascularization of the peripheral avascular retina, and recurrence of ROP.

If a fibrovascular proliferation reappeared with or without plus sign after the primary intravitreal anti-VEGF injection, we considered this to indicate a disease recurrence. Retreatment with laser photocoagulation or surgery based on the eye's situation was considered for these patients.

The infants were allocated into two groups. IVB group included patients who were treated with bevacizumab as initial treatment and the IVA group included patients who were treated with aflibercept as initial treatment.

The primary endpoints of the study were the rate of complete regression as well as the time to regression in each group. The recurrence rates as well as the median time to recurrence were compared between the groups as the secondary outcomes.

### Statistical analysis

To present data we used mean, standard deviation, median, range, frequency, and percentage. To compare results between two groups in subject variables we used t-test (for quantitative variable) and Chi-Square or Fisher exact test (for qualitative variable). When we compared eye variables between two groups we used Generalized Estimating Equation (GEE) to compensate possible correlation of the measurements. We report estimated median time to regression or recurrence by Kaplan Meier survival analysis in each group as an unbiased estimate whenever there were more than 50% events. To compare the survival rate between the two groups we used Log-rank test. All statistical analysis performed by SPSS software (IBM Corp. Released 2017. IBM SPSS Statistics for Windows, Version 25.0. Armonk, NY: IBM Corp.). A p-value less than 0.05 is considered statistically significant.

## Results

For this study, records of 920 eyes from 469 infants who received either intravitreal bevacizumab or aflibercept for ROP were reviewed. Thirty-one eyes were excluded due to incomplete documentation. A total of 889 eyes of 453 infants who were treated with either IVB or IVA were enrolled in the study. There were 865 eyes of 441 infants in the IVB group and 24 eyes of 12 infants in the IVA group. Infants received bilateral treatment in both groups with the exception of 17 infants (3.9%) in the IVB group who received unilateral treatment (p > 0.9).

The demographic data of the study groups are shown in Table 1. There were no significant differences between these two groups in terms of gestational age, birth weight, gender, co-morbidities (like sepsis, intraventricular hemorrhage (IVH)), transfusion, oxygen therapy, and intubation at the time of the treatment.

**Table 1:** Demographic data and characteristic findings in patients with retinopathy of prematurity received intravitreal Bevacizumab (IVB) vs. intravitreal Aflibercept (IVA)

Parameter	Level	Total	Type of Treatment		p
			IVB	IVA	
GA (weeks)	Mean ± SD	28.2 ± 2	28.2 ± 2	28.7 ± 2.3	0.230†
	Median (range)	28 (22 to 34)	28 (22 to 34)	28.5 (25 to 34)	
BW (g)	Mean ± SD	1121 ± 314	1119 ± 311	1205 ± 383	0.182†
	Median (range)	1060 (550 to 2700)	1050 (550 to 2700)	1190 (730 to 1975)	
Time of first treatment	Mean ± SD	59 ± 19	59 ± 19	55 ± 21	0.536¥
	Median	57	57	49	
Follow up (days)	Mean ± SD	285 ± 255	289 ± 257	143 ± 25	< 0.001¥
	Median	212	217	137	
Gender	Male	260	252 (57.2%)	8 (66.7%)	0.362*
	Female	193	189 (42.8%)	4 (33.3%)	
Twin	Yes	108	107 (24.3%)	1 (8.3%)	0.099*
	No	345	334 (75.7%)	11 (91.7%)	
O2 therapy	Yes	400	390 (88.4%)	10 (83.3%)	0.054**
	No	53	51 (11.6%)	2 (16.7%)	
Intubation	Yes	178	176 (40%)	2 (16.7%)	0.130*
	No	275	265 (60%)	10 (83.3%)	
Transfusion	Yes	257	247 (56%)	10 (83.3%)	0.077*
	No	196	194 (44%)	2 (16.7%)	
Intraventricular Hemorrhage	Yes	33	31 (7%)	2 (16.7%)	0.096**
	No	420	410 (93%)	10 (83.3%)	
Sepsis	Yes	177	173 (39.2%)	4 (33.3%)	0.673*
	No	276	268 (60.8%)	8 (66.7%)	
Phototherapy	Yes	297	293 (66.4%)	4 (33.3%)	< 0.001*
	No	156	148 (33.6%)	8 (66.7%)	
Anemia	Yes	46	39 (8.8%)	7 (58.3%)	< 0.001**
	No	407	402 (91.2%)	5 (41.7%)	
Acute respiratory distress syndrome	Yes	230	230 (52.2%)	0	< 0.001*
	No	223	21 1(47.8%)	12(100%)	
Laterality	Unilateral	17 (3.8%)	17 (3.9%)	0 (0.0%)	> 0.99**
	Bilateral	436 (96.2%)	424 (96.1%)	12 (100.0%)	
Eye	OD	443 (49.8%)	431 (49.8%)	12 (50.0%)	0.476¥
	OS	446 (50.2%)	434 (50.2%)	12 (50.0%)	
Zone pretreatment	1	197 (22.2%)	187 (21.6%)	10 (41.7%)	0.328¥
	2	692 (77.8%)	678 (78.4%)	14 (58.3%)	
Plus	Yes	885 (99.6%)	861 (99.5%)	24 (100.0%)	0.734¥
	No	4 (0.4%)	4 (0.5%)	0 (0.0%)	
Neovascularization of iris	Yes	58 (6.5%)	54 (6.2%)	4 (16.7%)	0.331¥
	No	831 (93.5%)	811 (93.8%)	20 (83.3%)	

† Based on t-test

* Based on Chi-Square test

** Based on Fisher exact test

¥ Based on GEE

Of the 865 eyes treated with IVB, 187 (21.6%) demonstrated zone I ROP and 678 (78.4%) demonstrated zone II ROP. Zone I and II of ROP were observed in 10 eyes (41.7%) and 14 eyes (58.3%) treated with IVA, respectively. The difference in the ROP zone was not statistically significant between the 2 treatment groups (p = 0.328).

Plus disease was seen in 861 eyes treated with bevacizumab (99.5%) and in 24 eyes treated with aflibercept (100%) (p = 0.734). Neovascularization of Iris (NVI) was observed in 54 (6.2%) eyes treated with bevacizumab and in 4 (16.7%) eyes treated with aflibercept (p = 0.331).

The mean age of the infants at the time of the injection was 59 ± 19 days and 55 ± 21 days in IVB and IVA groups, respectively (p = 0.536). Follow-up time was 289 ± 257 days in the IVB group and 143 ± 25 days in the IVA group (p < 0.001).

Table 2 shows the rate and the pattern of regression and recurrence in each group. All eyes in the IVA group showed initial regression of plus disease and resolution of ROP completely after the intravitreal injections. These regressions were achieved in 830 (96.0%) eyes that were injected with IVB. The regression rate was significantly more in the IVA group (p = 0.023). The median observed regression time was 10 days and 16 days in eyes treated with bevacizumab and aflibercept respectively. The Estimated time to have 50% of participants in IVB and IVA groups be regressed was 10 days (9.2 to 10.8) and 14 days (9.2 to 18.8), respectively.

**Table 2:** Comparison of regression and recurrence rate between patients with retinopathy of prematurity received intravitreal Bevacizumab (IVB) and intravitreal Aflibercept (IVA)

Parameter	Level	Total	Type of treatment		p‡
			IVB	IVA	
Regression	Number (%)	854 (96.1%)	830 (96.0%)	24 (100.0%)	0.023
	Estimated Median (days) (95% CI)†		10.0 (9.2 to 10.8)	14.0 (9.2 to 18.8)	
	Mean observed time (days) ± SD ¥		14 ± 10	22 ± 15	
	Median observed time (days) (range) ¥		10 (1 to 64)	16 (6 to 57)	
Recurrence	Number (%)	47 (5.5%)	34 (3.9%)	14 (58.3%)	< 0.001
	Estimated Median (days) (95% CI)†		NA	96.0 (76.3 to 115.7)	
	Mean observed time (days) ± SD ¥		47	104	
	Median observed time (days) ¥		23	92	

^‡^ Based on log-rank test

^†^ Estimated time to have 50% of participants in the group to have the outcomes

^¥^ Computed in the occurred participants

Recurrence was noted in 3.9% of eyes (34/865) in the IVB group and 58.3% of eyes (14/24) in the IVA group. Recurrence rates differed significantly between IVB and IVA groups (p < 0.001).

The time taken for 50% of the participants in the IVA group to have a recurrence after the initial regression was estimated to be 96 days (76.3 to 115.7).

No major ocular complications were detected related to intravitreal injections such as retinal detachment or endophthalmitis in each group. Post injection vitreous hemorrhage was reported in one eye among the IVB group. Subconjunctival hemorrhage was documented in a small number of infants that were limited to the injection site. These hemorrhages vanished during the first week following the intravitreal injection.

## Discussion

The use of anti-VEGF treatment for ROP is a safe method of treatment [12]. The findings presented herein revealed that although these two treatment groups having similar baseline demographic and clinical features, the regression was significantly more in the IVA group in comparison with the IVB group. The time of regression was longer in the IVA group (16 days vs.10 days). Despite initial favorable results with both bevacizumab and aflibercept injections for the treatment of type 1 ROP, there was significantly more recurrence in the IVA group in comparison with the IVB group.

Many studies have exhibited encouraging results obtained with intravitreal injections of anti-VEGF agents for the treatment of ROP. Among these drugs, bevacizumab is a widely applied agent for this purpose [6, 7, 13]. Bevacizumab provides faster regression of ROP than laser treatment and permits peripheral retinal vascularization with the benefit of being less destructive than laser treatment [14, 15]. It has been shown that more favorable refractive outcome can be achieved by anti-VEGF agents [2, 14, 15].

Although anti-VEGF agents can induce ROP regression efficiently, incomplete retinal vascularization may persist for a long period and may be a reason for recurrence. Therefore, after anti-VEGF injection long-term follow-ups may be required [2, 15].

Intravitreal injection of aflibercept was introduced by Salman et al. as an effective therapy for high-risk prethreshold type 1 ROP, with encouraging structural and functional results [16]. The systemic half-life of unbound aflibercept is lesser than bevacizumab (1.5 days vs. 20 days) and closer to that of ranibizumab (6 h). It has been shown that infants with ROP who were treated with IVB had a greater suppression of systemic VEGF than those who were treated with IVA [17].

In the present study, bevacizumab and aflibercept showed comparable activity in the early period, such as continued peripheral retinal vascularization. Although the regression was observed significantly more in the IVA group (100% vs. 96%, p = 0.023), this regression was achieved with more delay in the IVA group (16 days vs.10 days). Aflibercept has a greater binding capacity to VEGF than other anti-VEGF agents, also has a great affinity to PDGF. Furthermore, compared to bevacizumab, it has a longer period of intravitreal activity, which implies there's a higher risk of primary regression after injection [10]. Similar to our study, in a series of 46 eyes receiving intravitreal aflibercept monotherapy alone, regression of ROP was seen in 100% of eyes. Although 32.6% (15/46) of eyes reached complete vascularization, incomplete vascularization was persisted in 67.4% of the eyes [18].

These two agents displayed different activities in the infant’s eye during the follow-up period. Although recurrence was seen in both groups, it was later and more frequent in the aflibercept group. The rate of recurrence was 3.8% in the bevacizumab group which was comparable with that of the BEAT-ROP group [6]. Recurrence occurred in 58.3% of the aflibercept injected eyes. These dissimilarities may be justified by their different half-lives in human eyes, as the half-lives of bevacizumab and aflibercept are 4.9 and 7.13 days, respectively [19, 20]. As vascular endothelial growth factor (VEGF) is an essential angiogenic factor in both physiological and pathological conditions, therefore, more prolonged suppression of the VEGF agents may lead to a delay in normal peripheral vascularization of the avascular retina and more chance for recurrence of abnormal vessels [21]. In a recent study by Sukgen et al., treatment outcomes of intravitreal aflibercept were compared with ranibizumab. They showed that vascularization of the peripheral retina was achieved significantly later in the aflibercept group, they also explained this difference by their dissimilar half-lives in the vitreous. They concluded that due to the possibility of late recurrence, infants treated with aflibercept should be followed up for a longer period after injection [9].

The concurrence of ROP regression beside normal vasculogenesis is essential after ROP treatment, therefore the appropriate injection timing is an important point. Kim et al. [22] reported that due to the interaction of bevacizumab with normal retinal vascular development, the timing of anti-VEGF therapy should not be sooner than 30 weeks of post-coital age. In our study, both groups received anti-VEGF after 30 weeks based on post-coital age and there was no significant difference based on the time of the injection between the groups.

The mean recurrence time was at 47 days following the injection of bevacizumab and at 107 days after the injection of aflibercept in this investigation. Similar to our study, the recurrence was observed later in aflibercept injected eyes in comparison with bevacizumab injected eyes based on a recent study by Sukgen et al. (14.2 weeks vs. 8.2 weeks) [9]. A much higher rate of recurrence as well as later recurrence seen in the aflibercept group may be attributed to different pharmacokinetic features of this agent, including the longer half-life and longer clearance from vitreous body compared to bevacizumab [20].

Certainly, there are some limitations to this study. First, the number of cases between the groups is heterogeneous, although we partially compensated this heterogeneity by the statistical methods. Larger studies are required to confirm these results. Second, the presence of peripheral retinal avascular area was observed in both groups. However, our findings were solely based on indirect ophthalmoscopic observations. Thus, we cannot prohibit subtle vascular changes like faint leakages as have already been described with fluorescein angiography. Third, the dosing of aflibercept for intravitreal injection in neonates has not been thoroughly tested, and there is some debate about the most effective dose, which necessitates more investigations. Most of the previous studies consider half-dose aflibercept (1 mg/0.025 ml) as a standard dose for intravitreal injection in ROP [18, 23]. Recently, Ekinci et al. showed that 100% of eyes that had been treated with standard dosage were completely regressed and eyes with a lack of response were present only in the low-dose group (0.4 mg/0.01 ml), although the difference between the groups was not statistically significant.[23] Fourth, the study's retrospective nature resulted in varying follow-up periods and the possibility of confounding. Finally, the authors believe that further evidence is needed regarding the long-term effects of these anti-VEGF drugs on cardiological, nephrological, gastrointestinal, and neurodevelopmental development.

## Conclusion

Although the regression was significantly more in the IVA group (100% of the cases) but the recurrence rate was significantly more in this group, which may be due to the different pharmacokinetics of these drugs in the vitreous body. Further studies are needed to obtain ideal options for the treatment of ROP.

## Data Availability

The datasets used in the current study are available upon reasonable request.

## Abbreviations

ROP: Retinopathy of prematurity
IVB: Intravitreal bevacizumab
IVA: Intravitreal aflibercept
VEGF: Vascular endothelial growth factor
APROP: Aggressive posterior ROP
IVH: Intraventricular hemorrhage
